# Use of Cement Kiln Dust, Blast Furnace Slag and Marble Sludge in the Manufacture of Sustainable Artificial Aggregates by Means of Cold Bonding Pelletization

**DOI:** 10.3390/ma6083139

**Published:** 2013-07-25

**Authors:** Francesco Colangelo, Raffaele Cioffi

**Affiliations:** Department of Technology, University of Naples “Parthenope”, Centro Direzionale, Is. C4, Napoli 80143, Italy; E-Mail: rcioffi@uniparthenope.it

**Keywords:** cement kiln dust, granulated blast furnace slag, marble sludge, cold bonding pelletization process, sustainable artificial aggregates

## Abstract

In this work, three different samples of solid industrial wastes cement kiln dust (CKD), granulated blast furnace slag and marble sludge were employed in a cold bonding pelletization process for the sustainable production of artificial aggregates. The activating action of CKD components on the hydraulic behavior of the slag was explored by evaluating the neo-formed phases present in several hydrated pastes. Particularly, the influence of free CaO and sulfates amount in the two CKD samples on slag reactivity was evaluated. Cold bonded artificial aggregates were characterized by determining physical and mechanical properties of two selected size fractions of the granules for each studied mixture. Eighteen types of granules were employed in C28/35 concrete manufacture where coarser natural aggregate were substituted with the artificial ones. Finally, lightweight concretes were obtained, proving the suitability of the cold bonding pelletization process in artificial aggregate sustainable production.

## 1. Introduction

In 2010, the total amount of concrete used in Italy was 98.2 million cubic meters. The manufacture of these building materials required 186.6 million tons of aggregates, 222,000 tons of additives and about 40 million tons of cement. As the total amount of aggregate produced in Italy was about 300 million tons, the contribution of the concrete industry was equal to 62.5% with a consequent very high environmental impact [1].

Although in the last few years the production of concrete has slightly decreased, cement and natural aggregates consumption remains very high. In the former case, innovative binding systems as calcium sulfoaluminate cements or alkali activated binders can be found in literature [2,3,4,5,6,7,8,9,10,11,12,13,14,15], even if ordinary Portland cement (OPC) is still the preferred practical solution. OPC based binders are nowadays favored by a high diffusion of technological know-how and also by a huge experimental data collection throughout the previous century.

As far as the consumption of natural aggregates is concerned, the use of artificial and recycled aggregates as a partial replacement of natural ones has to be encouraged. In this context, the recycling of huge amounts of industrial solid wastes, sludges, reservoir sediments and demolition wastes in the production of concrete and aggregates is an important issue in directing waste management towards a more sustainable development [16,17,18,19,20,21,22,23,24,25,26,27,28,29,30,31,32,33].

In order to select the most suitable specific recycling process for each type of waste, great attention must be paid to their preliminary characterization. Chemical composition and particle size distribution are the properties that determine the best form of waste recovery as wastes may or may not have a binding behavior, depending on their content of hydraulically reactive oxides, while the specific surface area of powders may influence the reaction kinetic, workability/flowability, water demand, *etc*. [34,35,36].

Particles with very high specific surface area can be employed as a partial substitution either of the binder or of the finest fraction of aggregate. In the latter case they significantly influence the rheology of fresh mixtures allowing even the manufacture of special self-leveling mortars and self-compacting concrete, while in the former they can yield high-performance building components [37,38].

As far as the use of fine industrial wastes is concerned, cement kiln dust (CKD) and marble sludge (MS) represent two very interesting kinds of solid industrial wastes whose recycling needs to be improved. CKD is a very heterogeneous powder entrained in the combustion gasses flowing through the cement kiln and collected as residue in the air pollution control (APC) devices. Cement manufacturing process parameters, such as raw feeds, fuel characteristics and kiln technology largely influence the chemical composition and particle size of CKD. Because of the highly variable chemical composition of CKD, a number of research projects have been undertaken to mix this residue with different kinds of raw material, such as granulated blast furnace slag (GBFS), coal fly ash (FA) and silica fume (SF) in order to investigate its hydraulic capacity. Although CKD is most frequently recycled in the clinker kiln, limits on the amounts of alkalis and chlorides in cements call for further reuses of this waste to be explored [39,40,41,42].

The manufacture of artificial aggregates is one possible way of recycling CKD by mixing it with other fine hydraulic powders, as reported in a recent review by Maslehuddin *et al*., which reports an alternative application of CKD in the preparation of lightweight aggregates, concrete blocks, low-strength concrete and masonry cement [43].

As far as marble sludge is concerned, the extraction and processing of stone inevitably produces a huge mass of waste with a variety of different physical characteristics. The quarrying of stone yields blocks that usually do not account for more than 60% of the extracted material, with the consequent production of powdered waste causing damage to the environment. The recycling of these powders could offer many interesting advantages. Recent works have investigated the influence of marble powders on the rheological properties of cement pastes in the preparation of self-compacting concrete. Considering the specific characteristics of MS, further research on the effect of their addition to cementitious mixtures would certainly be worth considering [44,45].

The production of artificial aggregates may be accomplished by two different types of processes: cold-bonding cementitious pelletization and high temperature sintering. The latter technology has been extensively studied and the relevant literature is rich in applications for recycling waste materials such as fly ash and slag from coal combustion and from the incineration of solid wastes, metallurgical slags, cement kiln dust, tailings from mining and quarrying, sediments, *etc*. Unfortunately, very fine solid wastes may often have an unsuitable chemical composition for vitrification and sintering. So, artificial aggregate can be only obtained by granulation at low temperature of cementitious mixtures.

Over the last decade, the influence of pelletization process parameters and of the chemical and physical characteristics of powders on the physico-mechanical behavior of artificial aggregates has been studied by many authors. Baykal and Doven (2000) reported that optimum revolution plate speed should be chosen between 35 and 55 rpm and inclination angle between 35° and 55° in order to obtain good quality fly ash, lime and cement-based artificial aggregates [46]. More recently, Gesoglu *et al*. [47] investigated the effects of raw materials characteristics on the physico-mechanical properties of lightweight artificial aggregate (LWAA) produced via the cold bonding pelletization of fly ash, GBFS and cement mixtures. The authors conclude that maximum pelletization efficiency was reached with process parameters of 42 rpm and 45° for plate revolution speed and inclination, respectively [47]. In another study [48], blast furnace flue dust and basic oxygen furnace sludge were mixed with cement to prepare cold bonded pellets by means of a pelletizer disk with a rotating speed of 18 rpm and an inclination angle of 45°. After room curing, some of the pellets with a size fraction of 9.0–12.5 mm were selected and subjected to thermal analysis to investigate their thermal behavior.

The results of employing of GBFS cement-based cold bonded artificial aggregates in the manufacture of self-compacting concrete were also reported by [49]. This study concluded that the rounded shape and surface smoothness of the aggregate enhanced the viscosity and workability of concrete.

As far as chemically activated hydraulic systems are concerned, productions of cold bonded artificial aggregates in which Na_2_SO_4_ was employed to favor a greater formation of ettringite for increased strength were studied by [50]. Furthermore, alkaline activation solutions (NaOH and sodium silicate) were used by in the pelletization process to produce artificial aggregates making use of GBFS, FA and rice husk ash (RHA) cementitious mixtures [51]. In these systems, the presence of FA and RHA, favored the formation of geopolymeric phases and GBF addition yielded a calcium silicate hydrate (C–S–H) gel.

On the basis of previous studies by the authors of this paper [52,53,54], a preliminary study on the preparation of geopolymeric cold bonded artificial aggregates, where solid wastes, such as coal fly ash, blast furnace slag, clay reservoir sediment and municipal MSWI ash have been considered, is still in progress.

Currently, two industrial processes based on cold bonded cementitious mixtures are already applied to obtain commercial artificial aggregates: the Aardelite process, based on coal, fly ash and lime mixtures [55] and the Mapintec process, in which aggregate producing plants employ contaminated soils and cement-based mixtures [56]. These plants yield artificial aggregates whose technological properties have been widely tested in a number of civil engineering applications. In particular, the Mapintec technology has been applied as a soil stabilization/solidification treatment to produce non-hazardous aggregate employed in the in-situ remediation of contaminated sites.

In this paper the manufacture of artificial aggregates by means of a cold cementitious pelletization process is reported. CKD, MS and GBFS were employed as residues from industrial plants operating in Italy. The laboratory pelletization equipment makes use of a revolution plate with an inclination angle fixed at 45° and a rotating speed varying between 35 and 55 rpm. The hydration processes of the CKD/GBFS mixtures were studied by means of quantitative measurements of chemically bound water and evaluations of the mineralogical neo-formed phases. After 56-day curing, the pellets containing CKD, GBFS and MS were sieved and the main two fractions were tested to assess their physical-mechanical properties. Specifically, dry density, water absorption capacity and resistance to compression measurements were carried out. Finally, the best performance pellets were used to prepare strength class 28/35 concrete mixtures. After 28-day curing, cubic specimens, were tested from the technological point of view and compared with conventional concrete.

## 2. Materials and Experimental Program

### 2.1. Materials

#### 2.1.1. Chemical and Physical Characterization of Raw Materials

Two different types of cement kiln dust, named CKD_1_ and CKD_2_, a granulated blast furnace slag (GBFS) and a marble sludge (MS) were employed to produce cold-bonding pellets. The CKD_1_ and CKD_2_ samples were representative of plants operating in different conditions and were collected by electrostatic precipitators and baghouses, respectively. 

GBFS was added as a potential hydraulic binder whose latent reactivity can be activated by means of CKD oxides. The residues were characterised chemically, mineralogically and physically. 

A Perkin-Elmer Optima 2100 DV ICP-OES apparatus was employed to determine the samples chemical composition on a weighed amount of residue submitted to microwave digestion in a Perkin-Elmer Multiwave 3000 oven using a standard solution of HCl, HNO_3_ and HF followed by H_3_BO_3_ fluoride complexation. Chlorides and sulfates contents were determined by means of the Mohr method and ionic liquid chromatography, respectively. Franke method was used to determine Free CaO.

Table 1 reports chemical compositions of the employed industrial powdered waste. The CKD_1_ sample contained large amounts of calcite, expressed as high LoI and smaller amounts of free lime and dolomite [CaMg(CO_3_)_2_], whereas the CKD_2_ sample contained smaller amounts of calcite and higher amounts of free lime. This sample also contained significant amounts of anhydrite (CaSO_4_). 

It is well known that differences in terms of weight percentages of SO_3_, free-CaO and LoI should have a significant influence on the hydration of the hydraulic mixture.

The MS mainly consisted of CaCO_3_, while the chemical nature of GBFS was comparable to typical blast furnace slag composition.

**Table 1:** Chemical composition of materials (wt %).

Component	CKD_1_	CKD_2_	GBFS	MS
CaO	44.72	50.10	41.91	52.26
SiO_2_	16.35	12.10	35.16	1.12
Al_2_O_3_	4.95	4.26	10.76	0.37
Fe_2_O_3_	2.93	2.21	1.40	0.11
MgO	3.12	1.67	7.68	0.87
SO_3_	5.72	17.16	1.92	–
Na_2_O	0.93	1.26	0.11	0.14
K_2_O	4.90	5.83	0.14	0.10
Cl^−^	1.78	0.65	–	–
Alkali equivalent	4.02	4.92	0.18	–
Free-CaO	4.61	13.40	–	–
LoI *	23.30	9.16	1.78	40.74

* Loss on Ignition.

Fine powders were employed to identify the mineralogical phases present, making use of a Philips PW 1730 diffractometer. This apparatus used standard monochromatic CuKa radiation and operated at 40 kV and 20 mA. It can be seen that the slag consists mainly of a glassy phase.

**Figure 1:**
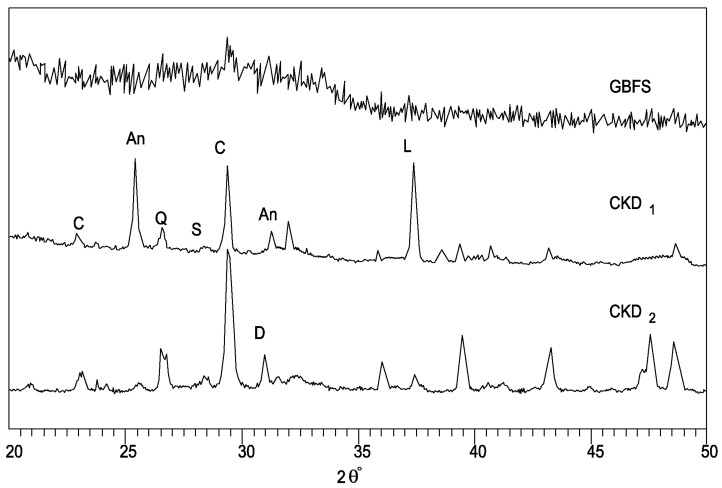
X-ray diffraction (XRD) analysis results for the two cement kiln dust (CKD) and granulated blast furnace slag (GBFS) samples (C = calcite, L = lime, D = dolomite, Q = quartz, An = anhydrite, S = sylvite).

Figure 1 shows the X-ray diffraction (XRD) analysis results for the two CKD and GBFS samples.

Results from the XRD analysis of the two CKD samples are shown in Figure 1. Calcite (CaCO_3_) was identified as the prevailing phase for CKD_2_ sample. It contains large amounts of calcite, expressed as high LoI, and small amounts of free lime [Ca(OH)_2_]. Traces of dolomite [CaMg(CO_3_)_2_] are also present. As the calcite is essentially inert, samples exhibiting high levels of carbonation and lacking free lime are considered less reactive. On the contrary, CKD_1_ contains smaller amounts of calcite and high amounts of free lime indicating that more Ca^2+^ will be available for a pozzolanic reaction. CKD_1_ also contains dolomite together with significant amounts of anhydrite (CaSO_4_). Finally, traces of sylvite (KCl) and significant amount of quartz are present in all the two samples. GBFS XRD trace indicates that glassy phases are mainly present [57,58].

**Figure 2:**
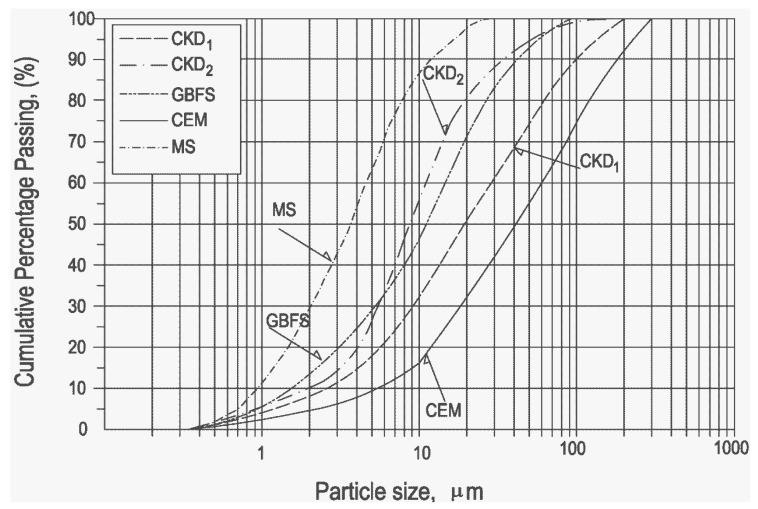
Particle size distribution of CKD_1_, CKD_2_ and GBFS.

Figure 2 shows the particle size distribution of the powders as measured through laser light scattering analysis by means of a Malvern’s Mastersizer 2000.

As for the CKD samples, the finest and most uniform was CKD_2_ with a specific surface area of 8270 cm^2^/g, a mean equivalent spherical particle diameter of 9 µm and 57% falling in the narrow range of 1–10 µm while 93% are smaller than 45 µm. On the other hand, 75% of the particles are in the range between 1 and 45 µm in the case of CKD_1_. In this case, a mean particle size of approximately 18 µm and a specific surface area of 6140 cm^2^/g were determined. CKD samples particle size distributions are compared with other raw materials of this experimental study. Marble sludge represents the finest component, with a specific surface area of 1.45 m^2^/g. Considering its very low reactivity it can be considered as the filling agent in aggregates. In Figure 2 CEM II 42.5 R (used for concrete mixes) and GBFS are also shown. These two components have a specific surface are of 3560 and 5530 cm^2^/g.

#### 2.1.2. Preparation and Characterization of Aggregates

CKD_1_ and CKD_2_, together with GBFS, were employed as binding agents in the preparation of pelletization mixtures. Furthermore, MS was added as filler to improve physical and mechanical properties of pellets. Two series of binding mixtures, named M1–M6 and M7–M12 contained different amounts of CKD_1_ and CKD_2_, respectively. In these series, GBFS to CKD ratios were 1, 2 and 4, respectively. The filler amount was fixed at 70%, in the mixtures M1–M3 and M7–M9, and 80% in the mixtures M4–M6 and M10–M12.

All the mixtures compositions are reported in Table 2.

**Table 2:** Pellets composition (wt %).

Mixtures	CKD_1_	CKD_2_	GBFS	MS
M1	15	–	15	70
M2	10	–	20	70
M3	6	–	24	70
M4	10	–	10	80
M5	6.5	–	13.5	80
M6	4.0	–	16.0	80
M7	–	15	15	70
M8	–	10	20	70
M9	–	6	24	70
M10	–	10	10	80
M11	–	6.5	13.5	80
M12	–	4.0	16.0	80

A laboratory-scale pelletization apparatus was employed for the manufacture of aggregates. It was equipped with a 90 cm diameter rotating disk whose tilting angle was fixed to 50°. The revolution speed was varied to optimize the pelletization process. The final settings were 35, 45 and 55 rpm. The powders were previously mixed with water by means of a Hobart mixer and then slowly and continuously poured onto the disk. The water to solid ratio (W/S) was adjusted using a nozzle to spray more water into the mixes. The optimum amount of water was fixed when pellets of the required size were formed. 

The prepared pellets were cured up to 56 days at room temperature and 100% RH. At the end of the curing phase the grain size distributions of the hardened granules were determined by sieving, according to UNI EN 933-1 standard. Two principal different size fractions were selected and tested. Specifically, granules of 4–12 mm and 12–20 mm in size were stored and submitted to physical and mechanical characterizations. In particular, the influence of revolution speed on physico-mechanical properties of pellets was studied. Specifically, the pellets were tested from technological point of view through the determination of: dry density, water absorption capacity (WAC) and compressive strength, according to UNI EN 1097-2:2008 and UNI EN 13055-1:2003 standards, respectively.

#### 2.1.3. Preparation and Characterization of Concrete.

Normal Strength Concrete C 28/35 (UNI EN 206–2006) mixes were designed by using eighteen types of pellets. In particular, two size classes, whose dimension varied in the ranges 4–12 mm and 12–20 mm, respectively, of the artificial aggregates M_1_, M_3_, M_4_, M_7_, M_9_ and M_10_ were employed as substitution of coarser natural ones. The aggregates were chosen considering the maximum amount of CKD (systems M1 and M7), the maximum amount of CKD + MS (systems M4 and M10) and highest compressive strength (systems M3 and M9). 

Finally, the total number (eighteen) of the aggregates types employed in concrete manufacturing was composed using, for each type, the pellets prepared with the three plate revolution speeds (35 rpm, 45 rpm and 55 rpm).

The mix-design of the concrete mixtures are shown in Table 3. 

**Table 3:** Concrete mixes compositions.

**Components**	**Concrete mixtures (kg/m^3^)**						
	CC	CM_1_	CM_3_	CM_4_	CM_7_	CM_9_	CM_10_
CEM II 42.5 R	310	310	310	310	310	310	310
Natural sand	708	708	708	708	708	708	708
NA 4–12 mm	699	–	–	–	–	–	–
NA 12–20 mm	501	–	–	–	–	–	–
AA 4–12 mm	–	498	490	511	506	516	509
AA 12–20 mm	–	353	348	361	358	366	361
Superplasticizer	3.6	5.7	5.1	4.5	4.7	4.4	4.5
W/C	0.5	0.5	0.5	0.5	0.5	0.5	0.5

In this table the compositions of the CM*_i_* concrete mixtures, containing the coarser artificial aggregates M_1_, M_3_, M_4_, M_7_, M_9_ and M_10_ are reported together with a benchmark Conventional Concrete (CC).

Eighteen concrete specimens were obtained using the above six artificial aggregates prepared at three different plate revolution speed values. 

Before concrete manufacturing, all the artificial aggregates were immersed in water up to saturation and then excessive surface water was eliminated by dripping. 

Specific amount of acrylic superplasticizer was added to each mixtures to guarantee the same consistency class S4 (UNI EN 206-1:2000) with a water to cement ratio (W/C) fixed to 0.5. 

28-day of water curing cubic specimens of 150 mm in size were cast and submitted to the density and compressive strength measurement (UNI EN 12390-3:2009).

Mean values and standard deviations of the experimental data were obtained by means of 9 replications.

### 2.2. Study of Binding Mixture Hydration Behavior

The binding mixtures, whose compositions are reported in Table 4, were characterized in terms of their hydraulic behavior. The six hydratory systems were cured with a water to solid ratio equal to 0.5. These systems have been designed with the same GBFS/CKD ratios (1:1, 2:1 and 4:1) considered for artificial aggregates in order to evaluate the differences hydration behavior by means of chemically bound water (see Table 2 and Table 4).

The curing conditions were: temperature 25 °C, relative humidity 100% and curing times of 1, 7, 28 and 56 days. Cylindrical paste specimens measuring 2 cm in diameter and 3 cm in height were prepared for each experimental condition. The hydration reactions of all the systems were stopped after each curing time by grinding the specimens under acetone, after which the powders were dried with diethyl ether. The hydration kinetic was measured by means of quantitative determinations of Chemically Bound Water (CBW) measured by mass loss at 750 °C.

**Table 4:** Hydratory systems composition (wt %).

System	CKD_1_	CKD_2_	GBFS
S1	50	–	50
S2	35	–	70
S3	20	–	80
S4	–	50	50
S5	–	35	70
S6	–	20	80

The quantitative determination of CBW content was performed as follows. A sample of mass *W*_0_ at time 0 acquires a mass *W_t_* after *t* days hydration. Of course, if *X*_0_ and *X_t_* are the fraction weight loss (at 750 °C) at time 0 and time *t*, respectively, it holds: *W*_0_(1 − X_0_) = *W*_t_(1 − *X_t_*). Then, *W*_0_ = *W_t_*(1 − *X_t_*)/(1 − *X*_0_). In conclusion, the percentage of CBW is: % CBW = (*W_t_* − *W*_0_)/*W*_0_ × 100. In this way, all the mix components that contribute to ignition loss are taken into account (CaCO_3_, Ca(OH)_2_, …).

The main mineralogical phases formed after 1, 7 and 28 days of curing were determined by means of X-ray diffraction analyses made on fine dried powders of each system. 

## 3. Results and Discussion 

### 3.1. Hydration Behaviour

**Figure 3:**
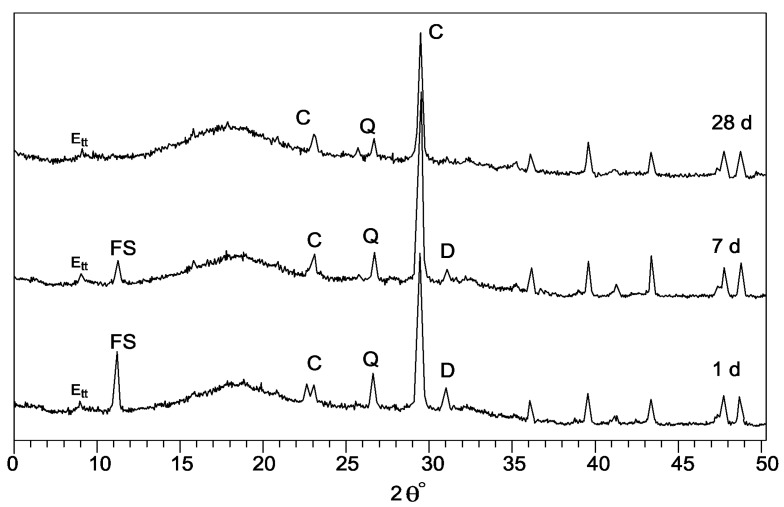
XRD spectra of hydratory system S1.

**Figure 4:**
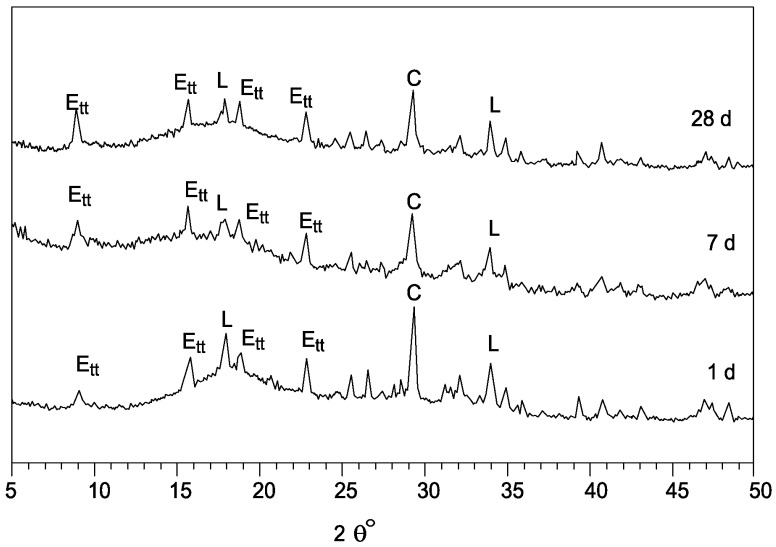
XRD spectra of hydratory system S4.

The results of XRD analyses carried out on the systems S1 and S4 are reported in Figure 3 and Figure 4, respectively. 

The XRD spectra related to the system S1 (Figure 3), containing CKD_1_ and GBFS, hydrated up to 28 days show the presence of the main peak for calcite at 29.4° 2θ that overlaps the well known broad diffusion peaks, at approximately 29.0° 2θ, assigned to calcium-silicate-hydrate (C–S–H) phases. In the same figure we can see that, as the reactivity of the CKD_1_ is a function of the alkali and free lime contents, a low conversion rate of residue is shown. This means that a limited availability of Ca^2+^ ions is possible only due to the presence of the negligible amounts of free lime (see Figure 1 and Table 1). It is well known that the carbonate particles of MS are inert, in fact, the calcite peaks remain almost constant during the hydration. Ettringite and an alumina, ferric oxide, monosulfate (AFm) phase were present after 1 day of hydration. Ettringite is converted to monosulphoaluminate when the sulfate source is not able to readily supply enough sulfate ions before the alumina content was completely hydrated. The AFm phase identified in the system S1 is the calcium monochloroaluminate hydrate, already known as Friedel’s salt, whose presence is the result of the high chloride content (1.78%, see Table 1) in the CKD_1_.

Starting from 7 days of curing, a decrease of the Friedel’s salt peak occurs due to the presence of sulphate ions. At 7-day curing the dolomite peaks (30.9° 2θ and 41.4° 2θ) decrease, while Ca(OH)_2_ peaks are not identified up to 28 days of hydration.

Figure 4 shows the diffractograms of the system S4, based on CKD_2_ and GBFS. During the early days of curing, ettringite was formed giving mixtures with very short setting time. After up to 28 days of curing the main phases were ettringite and Ca(OH)_2_ present.

In Table 5, the weight percentages of CBW measured on the hydrated systems are reported. The values are relative to 1, 7, 28 and 56 days of curing time.

Analysis of the values makes it possible to observe that a continuous rate is present when curing time increases. This is due to the continuous formation of the neo-formed hydration products associated with the slag activation by the CKDs. The amount of chemically bound water, present in the various forms of the main hydrated phases, such as C–S–H; AFm and ettringite, and the hydration kinetic vary considerably from a system to each other. 

**Table 5:** Chemically bound water of the systems hydrated for 1, 7, 28 and 56 days (wt %).

**System**	**Curing time (days)**			
	1	7	28	56
S1	0.5	1.8	4.5	5
S2	0.6	2	5	6
S3	0.9	2.5	7	9
S4	2.5	9	14	18
S5	4	11	16	20
S6	6	13	18	22

It can be seen that, in the case of the systems containing CKD_1_ (S1, S2 and S3), the cumulative amount of CBW is sensibly lower than that measured for the systems containing CKD_2_ (S4, S5 and S6). This is due to the fact that, in the case of CKD_1_, the amounts of lime and water-soluble alkali are not enough for the complete activation of slag. More precisely, the system S3, containing 80% of GBFS, show an amount of CBW lower than the system S4, containing 50% of GBFS. In the case of the slag activated by CKD_1_, the obtained hydration results are mainly due to the crystallization effect of the nuclei made by the almost inert CKD_1_ particles. In particular, the CBW amounts are almost the same for all the CKD_1_-based systems.

### 3.2. Aggregates Properties

#### 3.2.1. Effect of the Plate Revolution Speed

**Figure 5:**
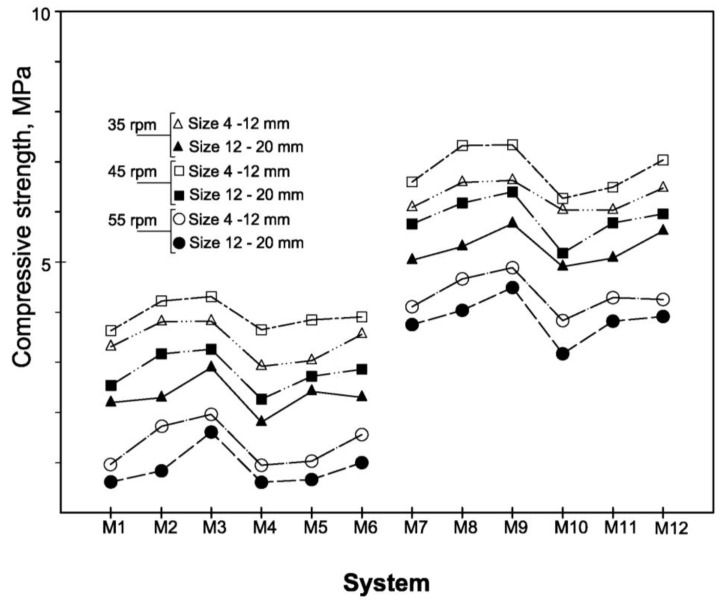
Compressive strength of the artificial aggregates after 56 days curing.

In Figure 5 are shown the values of compressive strength of the artificial aggregates evaluated after 56 days of curing. 

It is possible to observe that, for all the systems studied, the best values of strength are obtained with a plate revolution speed equal to 45 rpm. This result can be explained by considering that the increase of the revolution speed gives an increase of the coalescence process (collision and consolidation of the nuclei) of particles up to a limit value. Beyond this limit, the subsequent stratification of particles is counteracted by the centrifugal force that prevail on the gravitational component. This promotes adhesion of the particles on the side walls of the disc hindering the pelletization process. In fact, to obtain pellets of the same size at different speeds (35 rpm, 45 rpm and 55 rpm) feeding rate of the mixtures must be changed. In the case of highest rotation speed, the increase of the amount of mixture determine a decrease in capillary forces with a consequent greater degree of vacuum and minor mechanical performance of the pellets. The consequence of the previously described phenomena, is that, at each revolution speed, the coarser pellets (12–20 mm) have values of density and strength lower than those of smaller ones (4–12 mm). The differences are due to the combination through the collision and consolidation effects [46].

**Figure 6:**
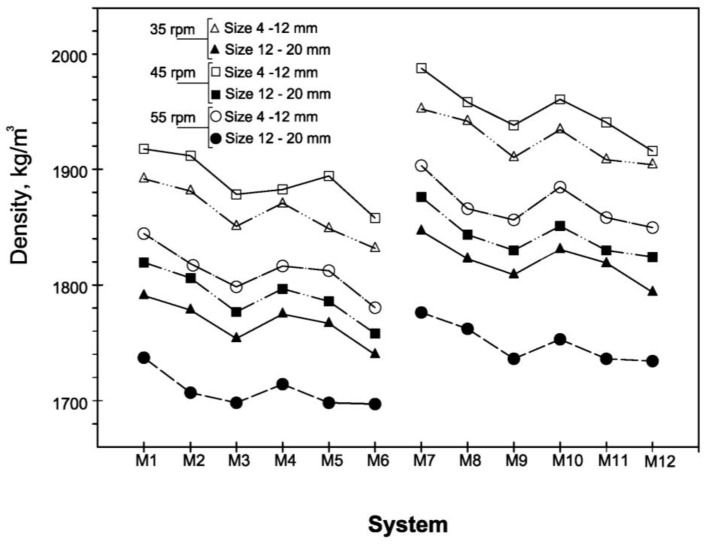
Dry density values of the artificial aggregates after 56 days curing.

**Figure 7:**
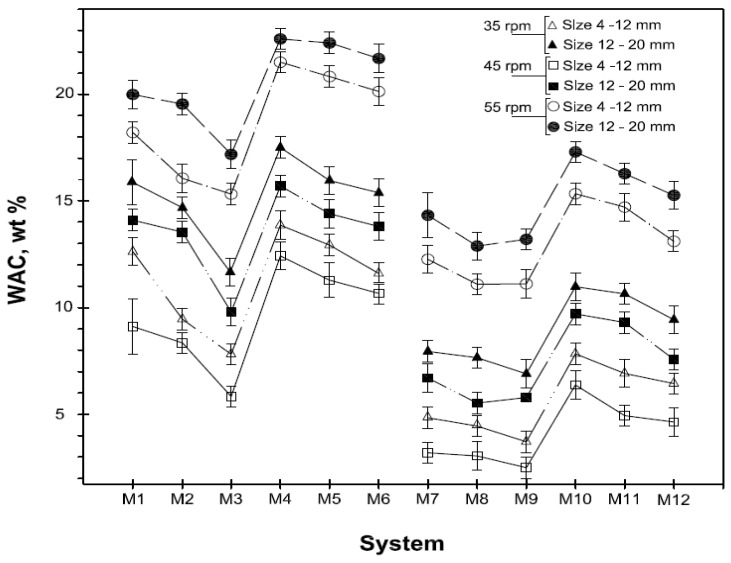
Water absorption capacity of the artificial aggregates after 56 days curing.

The general trend described for compressive strength was confirmed by the density and WAC values reported in Figure 6 and Figure 7. 

In fact, the pellets obtained with plate revolution speed equal to 45 rpm show highest density values and lowest percentage of water absorbed. Table 6 shows the influence of pelletization process parameters on physical and mechanical properties and turns out immediately the optimum process setting.

**Table 6:** Effect of pelletization process on physical and mechanical properties of aggregates.

Ranking	Compressive Strength	Density	WAC
1st	45 rpm/4–12 mm	45 rpm/4–12 mm	45 rpm/4–12 mm
2nd	35 rpm/4–12 mm	35 rpm/4–12 mm	35 rpm/4–12 mm
3rd	45 rpm/12–20 mm	55 rpm/4–12 mm	45 rpm/12–20 mm
4th	35 rpm/12–20 mm	45 rpm/12–20 mm	35 rpm/12–20 mm
5th	55 rpm/4–12 mm	35 rpm/12–20 mm	55 rpm/4–12 mm
6th	55 rpm/12–20 mm	55 rpm/12–20 mm	55 rpm/12–20 mm

#### 3.2.2. Effect of CKD, GBFS and MS

The effect of the different chemical compositions of the two CKDs employed is evident by observing the variation existing between the measured physical and mechanical properties of the pellets. In fact, the mixtures containing CKD_1_ (types M_1_–M_6_) exhibit strength values lower than those containing CKD_2_ (types M_7_–M_12_), in all case for the same plate revolution speed. In particular, the best systems with CKD_2_ (types M_8_ and M_9_) show a strength about 64% higher than system M_3_, that is the best mixtures containing CKD_1_. In the case of less resistant systems, the worst prepared with CKD_2_ (type M_10_) is about three times higher than the system M_4_.

**Figure 8:**
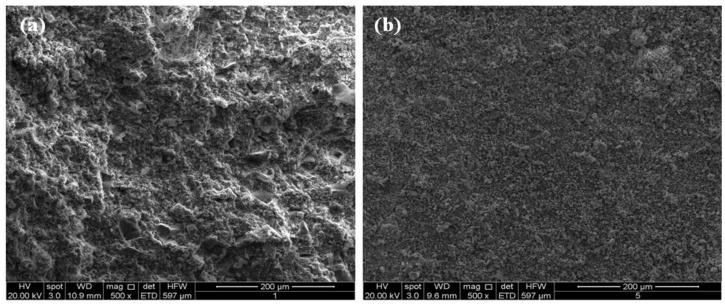
SEM micrographs (500×) of the systems M1 (**a**) and M7 (**b**) after 28-day curing.

**Figure 9:**
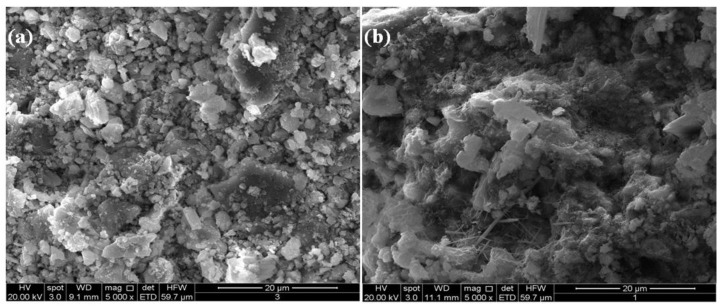
SEM micrographs (5000×) of the systems M1 (**a**) and M7 (**b**) after 14-day curing.

**Figure 10:**
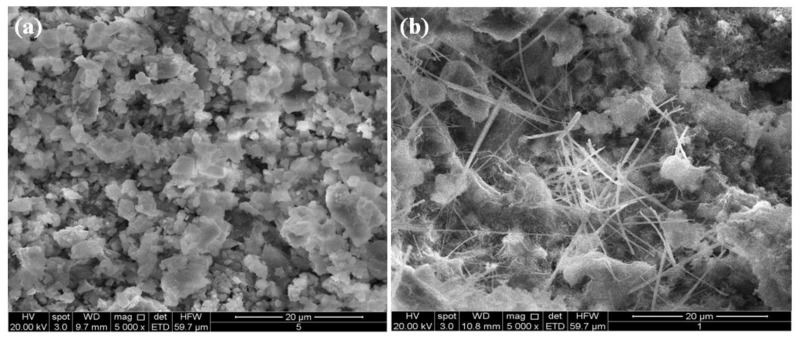
SEM micrographs (5000×) of the systems M1 (**a**) and M7 (**b**) after 28-day curing.

If we compare strength measured for each type of system, the variation of the plate revolution speed gives maximum decreases of about 58% and 90%, in the cases of mixtures containing CKD_2_ and CKD_1_, respectively. These differences have to be associated to the chemical nature of CKD samples. In fact, in the case of mixtures M_1_–M_6_, due to low activation action of CKD_1_ on GBFS hydration, they develop minor amount of neo-formed phases (see also Figure 8, Figure 9 and Figure 10) with consequent greater effect of centrifugal force acting on powdered particles which are less bound by the hydration products.

The absolute values of strength varied between 4.4 MPa and 0.6 MPa for the systems with CKD_1_ and between 7.4 MPa and 3.2 MPa for the systems with CKD_2_.

The influence of CKD amount can be studied looking at the strength differences existing between the systems M_1_–M_3_ and between the systems M_7_–M_9_. In each of these two series, the CKD/GBFS ratios are 1.0, 0.5 and 0.25, respectively. The strength measured on coarser pellets of types M_1_–M_3_, prepared with plate revolution speed of 45 rpm, vary from a minimum of 3.6 MPa to a maximum of 4.4 MPa, with an increase of about 21%. While, for the systems M_7_–M_9_, prepared with the same operative conditions, an increase of about 12% is observed. In fact, the strength passed from 6.6 MPa to 7.4 MPa. These results agree, once more, with the difference in reactivity showed by the two CKD samples employed. The better activating capacity of CKD_2_ gives to the system M_7_, containing only 15% of GBFS, a very good hydraulic capacity in comparison with that showed from the system M_9_. 

Less evident differences in terms of mechanical behavior were detected at different CKD/GBFS ratios for the systems containing higher quantity of very fine MS. In fact, the coarser aggregates prepared at 45 rpm have strength in a range of 3.7–3.9 MPa, for the series M_4_–M_6_, while, in the same conditions, values 6.4 MPa and 6.9 MPa are observed for the series M_10_–M_12_. In any case, all the systems with 80% of MS show a lower strength than those containing 70% of MS.

As far as the pellets density is of concerned, results of great interest were also determined. The higher values, measured on pellets prepared with a plate revolution speed of 45 rpm, range between 1918 and 1857 kg/m^3^ and 1984 and 1928 kg/m^3^ for the pellets containing CKD_1_ and CKD_2_, respectively. While the minimum values of density, relative to the pellets obtained with a plate revolution speed of 55 rpm, are 1696 kg/m^3^ and 1732 kg/m^3^ for the systems containing CKD_1_ and CKD_2_, respectively.

All the results are such that the pellets produced in this work can be classified as lightweight aggregate, according to UNI EN 13055-1:2005 standard where the density value required for such classification must lie between 0.50 and 2.00 g/cm^3^. Even in most of the artificial aggregates can be considered lightweight according to UNI EN 13055, some of them seem very close to standard limit. So, it can be argued that in a larger scale production, mixtures compositions and pelletization process parameters should be carefully fixed.

The filler effect of very fine MS particles is evident if we compare the density of the pellets prepared with 80% of MS (types M_4_–M_6_ and M_10_–M_12_) with those containing 70% MS (types M_1_–M_3_ and M_7_–M_9_). In these cases, systems with 80% MS had lower density comparing systems with the same CKD/GBFS ratio, plate revolution speed and grain size.

In addition, differences of the same order of magnitude are observed even if the behaviour of pellets with different particle sizes are compared. In fact, the trends described remain the same for both pellets with dimensions in the range 4–12 mm and those in the range 12–20 mm.

Figure 8, Figure 9 and Figure 10 showed the results of SEM analysis made on the M1 and M7 hardened systems. Figure 8a,b report micrographs, at a low magnification (500×), of systems M_1_ and M_7_ both cured for 28 days. In these figures, samples with different density are shown. In particular, the M_7_ system (Figure 8b) shows a more compact microstructure in comparison to M_1_ one (Figure 8a).

These findings agree with the results of the mineralogical analysis above described. In fact, flaky calcium silicate hydrate (C–S–H) crystals and needle-like ettringite crystals are more evident in higher magnification (5000×) micrographs showed in the Figure 9b and Figure 10b. 

In the Figure 9b and Figure 10b the microstructure of M_1_ system revealed to be more hydrated than M7 system both after 14 and 28 days curing.

### 3.3. Concrete Characterization

Table 7 reports the results of physical and mechanical tests on concrete. Particularly, density and compressive strength values, obtained by testing of concrete specimens after 28 days of curing, are shown. 

**Table 7:** Physical and mechanical characterization of concrete according to UNI EN 206-1.

Concrete	Strength (MPa)	Strength class UNI EN 206-1	Density [kg/m^3^]	Density class UNI EN 206-1
CC	37.3 ± 1.6	30/33 (Not LC)	2245.8 ± 62	Not LC
CM_1-45_	22.5 ± 1.2	LC 20/22	1865.5 ± 41	D2.0
CM_3-45_	27.4 ± 1.1	LC 20/22	1897.3 ± 44	D2.0
CM_4-45_	22.6 ± 0.8	LC 20/22	1876.7 ± 50	D2.0
CM_7-45_	38.8 ± 1.0	LC 35/38	1927.9 ± 32	D2.0
CM_9-45_	41.8 ± 1.7	LC 35/38	1981.4 ± 71	D2.0
CM_10-45_	40.5 ± 1.5	LC 35/38	1938.6 ± 87	D2.0
CM_1-35_	18.9 ± 0.8	LC 16/18	1779.3 ± 22	D1.6
CM_3-35_	22.8 ± 0.9	LC 20/22	1797.2 ± 69	D1.6
CM_4-35_	17.4 ± 0.6	LC 12/13	1782.1 ± 51	D1.6
CM_7-35_	36.9 ± 1.2	LC 30/33	1912.8 ± 42	D2.0
CM_9-35_	39.6 ± 1.2	LC 35/38	1943.8 ± 88	D2.0
CM_10-35_	36.0 ± 0.9	LC 30/33	1924.8 ± 67	D2.0
CM_1-55_	7.2 ± 0.3	Nc	1750.1 ± 22	D1.6
CM_3-55_	11.4 ± 0.4	LC 8/9	1779.6 ± 43	D1.6
CM_4-55_	7.3 ± 0.4	Nc	1768.3 ± 52	D1.6
CM_7-55_	24.9 ± 0.5	LC 20/22	1854.5 ± 38	D2.0
CM_9-55_	28.8 ± 1.2	LC 25/28	1862.9 ± 62	D2.0
CM_10-55_	23.1 ± 0.9	LC 20/22	1879.2 ± 38	D2.0

CM*_x-y_*: means concrete made using AA prepared with the mixtures M*_x_* and a plate revolution speed of *y* rpm; Nc: strength not classified.

The strength measured for CM_i_ specimens are in the ranges 22.5–41.8 MPa, 17.4–39.6 MPa and 7.2–28.8 MPa for the concrete containing artificial aggregates (AAs) produced with a plate revolution speed of 45, 35 and 55 rpm, respectively. As expected, the trend of the results is in line with that observed for the pellets. 

As the design of mixtures was aimed to obtain C28/35 strength class, the results of CMs specimens characterization were used to investigate the influence of cold-bonded AAs addition on physical and mechanical properties of the non-conventional concrete.

The data reported in table 7 show that, after 28 days of curing, the strength of the stronger concrete containing AAs prepared at 45 rpm reaches values higher 12% than those measured for the reference one. In the case of concrete containing pellets produced at 35 rpm the maximum strength value is 6.2% higher than that of CC. Finally, the stronger concrete containing AAs obtained at 55 rpm shows strength 22.8% less than the conventional one.

In any case, the highest strength is always showed by the concrete containing M_9_ type aggregate. This mixture was made with 6% of CKD_2_, 70% of MS and 24% of GBFS (see Table 3).

In Table 7 it can also be seen that all the specimens have density values <2000 kg/m^3^ that is the limit indicated in UNI EN 206-1:2006 standard for lightweight structural concrete (LC). According to this standard, all the CM concrete prepared at 45 rpm, together with those containing CKD2 and produced at 35 and 55 rpm can be classified as D 2.0 concrete (density between 1800 and 2000 kg/m^3^), while all the others are classified as D 1.8 (density between 1600 and 1800 kg/m^3^).

In addition, as far compressive strength is concerned, the above standard classifies lightweight concretes in eleven strength classes from LC 8/9 to LC 55/60, where LC *X*/*Y* type means that at least 95% of strength determinations are greater than *X* MPa for cylindrical samples 100 mm in diameter and 150 mm in height or greater than *Y* MPa for cubic samples 150 mm in size. 

In Table 7, each CM was associated to a specific strength class. It can be seen that the best performances are showed by the three concretes containing granules made using the more reactive CKD_2_ samples and with plate revolution speed of 45 rpm plus one concrete with AAs M9 and at 35 rpm. All the data specifically show that the concretes containing AA made with CKD_1_ at 45 rpm and those containing M_4-35_, M_7-55_ and M_10-55_ AAs belong to the class LC 20/22. The concretes containing AAs made with CKD_2_ at 45 rpm show the best compressive strength and belong to the class LC 35/38 together with that prepared with type AA M_9-35_. Concrete CM_9-55_ can be associate to the strength class LC 25/28 while CM_9-55_ and CM_9-55_ specimens belong to the class LC 30/33. Finally, the strength showed by the CM_1-35_, CM_4-35_ and CM_3-55_ concretes allow to associate these mixtures to the strength class LC 16/18, LC 12/13 and LC 8/9, respectively. The worst strength are observed for the concrete CM_1-55_ and CM_4-55_ that shown 7.2 and 7.3 MPa, respectively. These concretes are not classified by the UNI EN 206-1 standard. 

## 4. Conclusions

At the end of all experimental tests the following conclusions can be drawn:

1. Mixtures containing cement kiln dust, blast furnace slag and marble sludge were used in the manufacture of cold bonded lightweight artificial aggregates, which can be employed in the preparation of structural lightweight concrete. Two different CKD samples have been employed in order to take into account the heterogeneity between two plants operating under different conditions.
2. The pellets were produced making use of a *easily scalable* from *pilot* to process-*scale* apparatus based on a rotating and tilting plate. Starting from some experimental results present in literature and previous studies of the authors, a wide investigation range was fixed.
3. Best mechanical performance of artificial aggregates was achieved for a plate revolution speed of 45 rpm and a tilt angle of 50°. In any case, depending on the CKD chemical composition, aggregates produced at 35 rpm and 55 rpm also showed adequate physical and mechanical performances.
4. Almost all the lightweight concrete specimens containing the artificial aggregates prepared in this work were able to satisfy the technical requirements in force in Italy for structural use. Furthermore, artificial aggregates physico-mechanical properties have a relevant influence on concrete specimens performances.
5. Assessment by technical committees of all physical, mechanical and environmental properties (of both granules and concretes) is necessary before the use of novel artificial aggregate in concrete manufacture. The sustainability of the whole proposed process needs to be quantified by means of appropriate evaluation of specific indicators such as those used in Life Cycle Assessment (LCA) and Life Cycle Cost (LCC) methodologies [59].

## References

[1] ATECAP: Italian Premixed Concrete Technological and Economical Association, Annual Report. http://www.atecap.com.

[2] Andini S., Cioffi R., Colangelo F., Grieco T., Montagnaro F., Santoro L. (2008). Coal fly ash as raw material for manufacture of geopolymer-based product. Waste Manag..

[3] Beretka J., Cioffi R., Marroccoli M., Valenti G.L. (1996). Energy-saving cements obtained from chemical gypsum and other industrial wastes. Waste Manag..

[4] Bignozzi M.C., Manzi S., Lancellotti I., Kamseu E., Barbieri L., Leonelli C. (2013). Mix-design and characterization of alkali activated materials based on metakaolin and ladle slag. Appl. Clay Sci..

[5] D’Auria I., Lamberti M., Mazzeo M., Milione S., Roviello G., Pellecchia C. (2012). Coordination Chemistry and Reactivity of Zinc Complexes Supported by a Phosphido Pincer Ligand. Chem. Eur. J..

[6] Duxson P., Fernández-Jiménez A., Provis J.L., Lukey G.C., Palomo A., van Deventer J.S.J. (2007). Geopolymer technology: The current state of the art. J. Mater. Sci..

[7] Ferone C., Colangelo F., Roviello G., Asprone D., Menna C., Balsamo A., Prota A., Cioffi R., Manfredi G. (2013). Application-oriented chemical optimization of a metakaolin based geopolymer. Materials.

[8] Ferone C., Roviello G., Colangelo F., Cioffi R., Tarallo O. (2013). Novel hybrid organic geopolymer materials. Appl. Clay Sci..

[9] Menna C., Asprone D., Ferone C., Colangelo F., Balsamo A., Prota A., Cioffi R., Manfredi G. (2012). Use of geopolymers for composite external reinforcement of RC members. Compos. B Eng..

[10] Flatt R.J., Roussel N., Cheeseman C.R. (2012). Concrete: An eco material that needs to be improved. J. Eur. Ceram. Soc..

[11] Pacheco-Torgal F., Castro-Gomes J., Jalali S. (2008). Alkali-activated binders: A review: Part 1. Historical background, terminology, reaction mechanisms and hydration products. Constr. Build. Mater..

[12] Pera J., Ambroise J. (2004). New applications of calcium sulfoaluminate cement. Cem. Concr. Res..

[13] Peysson S., Péra J., Chabannet M. (2005). Immobilization of heavy metals by calcium sulfoaluminate cement. Cem. Concr. Res..

[14] Roviello A., Buono A., Carella A., Roviello G., Cassinese A., Barra M., Biasucci M. (2007). Regioregular poly[3-(4-alkoxyphenyl)thiophene]s. J. Polym. Sci. A Polym. Chem..

[15] Wu K., Shi H., Guo X. (2011). Utilization of municipal solid waste incineration fly ash for sulfoaluminate cement clinker production. Waste Manag..

[16] Bignozzi M.C., Saccani A. (2012). Ceramic waste as aggregate and supplementary cementing material: A combined action to contrast alkali silica reaction (ASR). Cem. Concr. Compos..

[17] Cheeseman C.R., Makinde A., Bethanis S. (2005). Properties of lightweight aggregate produced by rapid sintering of incinerator bottom ash. Resour. Conserv. Recycl..

[18] Chen H.J., Yang M.D., Tang C.W., Wang S.Y. (2012). Producing synthetic lightweight aggregates from reservoir sediments. Constr. Build. Mater..

[19] Cioffi R., Colangelo F., Montagnaro F., Santoro L. (2011). Manufacture of artificial aggregate using MSWI bottom ash. Waste Manag..

[20] Colangelo F., Cioffi R., Montagnaro F., Santoro L. (2012). Soluble salt removal from MSWI fly ash and its stabilization for safer disposal and recovery as road basement material. Waste Manag..

[21] Colangelo F., Cioffi R., Lavorgna M., Verdolotti L., de Stefano L. (2011). Treatment and recycling of asbestos-cement containing waste. J. Hazard. Mater..

[22] Andini S., Montagnaro F., Santoro L., Accardo G., Cioffi R., Colangelo F. (2013). Mechanochemical processing of blast furnace slag for its reuse as adsorbent. Chem. Eng. Trans..

[23] Limbachiya M.C. (2010). Recycled aggregates: Production, properties and value-added sustainable applications. J. Wuhan Univ. Technol. Mater. Sci..

[24] Limbachiya M.C., Leelawat T., Dhir R.K. (2000). Use of recycled concrete aggregate in high, strength concrete. Mater. Struct..

[25] Limbachiya M.C., Meddah M.S., Ouchagour Y. (2012). Use of recycled concrete aggregate in fly ash concrete. Constr. Build. Mater..

[26] Manzi S., Mazzotti C., Bignozzi M.C. (2013). Short and long-term behaviour of structural concrete with recycled concrete aggregate. Cem. Concr. Compos..

[27] Poon C.S., Kou S.C., Lam L. (2002). Use of recycled aggregates in molded concrete bricks and blocks. Constr. Build. Mater..

[28] Saccani A., Bignozzi M.C. (2010). ASR expansion behavior of recycled glass fine aggregates in concrete. Cem. Concr. Res..

[29] Sagoe-Crentsil K.K., Brown T., Taylor A.H. (2001). Performance of concrete made with commercially produced coarse recycled concrete aggregate. Cem. Concr. Res..

[30] Saikia N., de Brito J. (2012). Use of plastic waste as aggregate in cement mortar and concrete preparation: A review. Constr. Build. Mater..

[31] Sani D., Moriconi G., Fava G., Corinaldesi V. (2005). Leaching and mechanical behaviour of concrete manufactured with recycled aggregates. Waste Manag..

[32] Topcu I.B., Sengel S. (2004). Properties of concretes produced with waste concrete aggregate. Cem. Concr. Res..

[33] Wainwright P.J., Cresswell D.J.F. (2001). Synthetic aggregates from combustion ashes using an innovative rotary kiln. Waste Manag..

[34] De Schutter G., Taerwe L. (1995). General hydration model for Portland cement and blast furnace slag cement. Cem. Concr. Res..

[35] Uchikawa H., Uchida S., Ogawa K., Hanehara S. (1984). Influence of CaSO_4_·2H_2_O, CaSO_4_·12H_2_O and CaSO_4_ on the initial hydration of clinker having different burning degree. Cem. Concr. Res..

[36] Vuk T., Tinta V., Gabrovšek R., Kaučič V. (2001). The effects of limestone addition, clinker type and fineness on properties of Portland cement. Cem. Concr. Res..

[37] Bignozzi M.C., Sandrolini F. (2006). Tyre rubber waste recycling in self-compacting concrete. Cem. Concr. Res..

[38] Felekoglu B. (2007). Utilisation of high volumes of limestone quarry wastes in concrete industry (self compacting concrete case). Resour. Conserv. Recycl..

[39] Corish A., Coleman T. (1995). Cement kiln dust. Concrete.

[40] Haynes B.W., Kramer G.W. (1982). Characterization of U. S. Cement Kiln Dust.

[41] Huntzinger D.N., Gierke J.S., Sutter L.L., Kawatra S.K., Eisele T.C. (2009). Carbon dioxide sequestration in cement kiln dust through mineral carbonation. Environ. Sci. Technol..

[42] Rafat S. (2008). Cement Kiln Dust. Waste Materials and By-Products in Concrete.

[43] Maslehuddin M., Al-Amoudi O.S.B., Shameem M., Rehman M.K., Ibrahim M. (2008). Usage of cement kiln dust in cement products—Research review and preliminary investigations. Constr. Build. Mater..

[44] Alyamaç K.E., Ince R. (2009). A preliminary concrete mix design for SCC with marble powders. Constr. Build. Mater..

[45] Cioffi R., Colangelo F., Santoro L. Durability of Mortars Prepared with Innovative Eco Compatible Binders. 11DBMC International Conference on Durability of Building Materials and Components.

[46] Baykal G., Döven A.G. (2000). Utilization of fly ash by pelletization process; theory, application areas and research results. Resour. Conserv. Recycl..

[47] Gesoglu M., Güneyisi E., Öz H.Ö. (2012). Properties of lightweight aggregates produced with cold-bonding pelletization of fly ash and ground granulated blast furnace slag. Mater. Struct..

[48] Su F., Lampinen H.O., Robinson R. (2004). Recycling of sludge and dust to the BOF converter by cold bonded pelletizing. ISIJ Int..

[49] Güneyisi E., Gesoğlu M., Booya E. (2012). Fresh properties of self-compacting cold bonded fly ash lightweight aggregate concrete with different mineral admixtures. Mater. Struct..

[50] Geetha S., Ramamurthy K. (2010). Environmental friendly technology of cold-bonded bottom ash aggregate manufacture through chemical activation. J. Clean. Prod..

[51] Bui L.A.T., Hwang C.L., Chen C.T., Lin K.L., Hsieh M.Y. (2012). Manufacture and performance of cold bonded lightweight aggregate using alkaline activators for high performance concrete. Constr. Build. Mater..

[52] Andini S., Cioffi R., Colangelo F., Ferone C., Montagnaro F., Santoro L. (2011). Characterization of geopolymer materials containing MSWI fly ash and coal fly ash. Adv. Sci. Technol..

[53] Ferone C., Colangelo F., Cioffi R., Montagnaro F., Santoro L. (2011). Mechanical performances of weathered coal fly ash based geopolymer bricks. Procedia Eng..

[54] Ferone C., Colangelo F., Cioffi R., Montagnaro F., Santoro L. (2013). Use of reservoir clay sediments as raw materials for geopolymer binders. Adv. Appl. Ceram..

[55] Aarding Lightweight Granulates BV Home Page. http://www.aardinglg.com/.

[56] MAPINTEC Home Page. http://www.mapintec.it/.

[57] Ballester P., Mármol I., Morales J., Sánchez L. (2007). Use of limestone obtained from waste of the mussel cannery industry for the production of mortars. Cem. Concr. Res..

[58] Sugrañez R., Álvarez J.I., Cruz-Yusta M., Mármol I., Morales J., Sánchez L. (2013). Controlling microstructure in cement based mortars by adjusting the particle size distribution of the raw materials. Constr. Build. Mater..

[59] Colangelo F., Vaccaro R., Cioffi R. Life Cycle Assessment of Sustainable Concrete Made with Recycled Aggregates. II International Conference on Sustainable Construction Materials and Technology.

